# Phenotypic Plasticity of Early and Late Successional Forbs in Response to Shifts in Resources

**DOI:** 10.1371/journal.pone.0050304

**Published:** 2012-11-21

**Authors:** Yingxin Huang, Xueyong Zhao, Daowei Zhou, Hongxiang Zhang, Wei Zheng

**Affiliations:** 1 Northeast Institute of Geography and Agroecology, Chinese Academy of Sciences, Changchun, China; 2 Cold and Arid Regions Environmental and Engineering Research Institute, Chinese Academy of Sciences, Lanzhou, China; University of Nottingham, United Kingdom

## Abstract

We compared the phenotypic plasticity of two early successional forbs of nutrient-poor mobile dunes (*Agriophyllum squarrosum* and *Corispermum macrocarpum*) and two later successional forbs (weeds) of stabilized, higher nutrient dunes and cropland (*Chenopodium acuminatum* and *Salsola collina*) to variations in environmental factors. A controlled (including soil nutrients, water, and population density) greenhouse experiment was conducted in Horqin sandy land, China. Late successional species had high plasticity in growth response to nutrients and water or high performance in high soil nutrients and water, reflecting their higher nutrient habitat. In contrast, the early successional species have low plasticity, reflecting their adaptation to resource-poor early successional soil. Late successional species did not always have higher reproductive effort than early successional species. Plants did not have a uniform strategy of increasing reproductive effort with any environmental stressors. Reproductive effort increased with increasing water availability and decreasing nutrient levels, while density had no effect. Patterns of plasticity traits for late successional species exhibited a complex of Master-of-some and Jack-of-all-trades. Late successional species had higher performance or higher plasticity than early successional species.

## Introduction

Phenotypic plasticity is usually defined as the ability of an individual genotype to modify its growth and development when exposed to different environmental conditions [1]–[3]. As an important strategy for plants to maximize or maintain fitness in variable biotic and abiotic environments [4], many ecologists have embraced the idea that under many circumstances such phenotypic plasticity can be adaptive [5], [6]. Phenotypic plasticity allows individuals to exhibit advantageous phenotypes in a breadth of environmental conditions [7].

High plasticity is also commonly invoked to explain invasion success [8]. Invasive species may have increased plasticity relative to non-invasive species [9]–[11]. Richards et al. (2006) summarizes that the invader may benefit from three idealized scenarios: (1) a Jack-of-all-trades, in which invaders maintain fitness in unfavorable environments; (2) a Master-of-some, in which invaders increase fitness in favorable environments; or (3) a Jack-and-master, in which invaders combine some level of both above scenarios. Support for the scenarios is found in later studies [12]–[14].

In succession, early successional species colonize the disturbed site. As colonizers replace indigenous communities, and late successional species replace early successional species. The late successional species have some of the same advantages as invaders. Therefore, under some conditions late successional species prevail in competition with early successional species leading to a shift in community composition. There are different patterns of phenotypic plasticity between early and late successional species. Some studies point out that early successional species showed higher photosynthetic plasticity than late successional species due to selection pressures [15], [16]. However it is also found that the late successional species have higher phenotypic plasticity than early successional species. For example, shade tolerant, late successional species exhibit high morphological plasticity [17], [18], and late successional forbs have stronger plastic responses to nutrient patchiness compared to mid-successional forbs [19]. Additional studies comparing phenotypic plasticity between different successional species are necessary to understand patterns of response to successional environments.

Morphological traits are important to resource partitioning in many plants [20]–[22]. Reproductive effort, which shows the proportion of the plant's resources allocated to reproductive activities, is an important component of plant fitness [23]–[25]. Reproductive effort of plants varies across different seral environments by the adjusting partitioning of resources and morphological traits (in relation to reproductive fitness). Evolutionary selection should favor different patterns of reproductive effort during early or late stages of ecological succession [26]. Invasive species showed higher reproductive effort that varies across a range of resource levels [27], [28]. Recent studies have suggested that invasive or late successional species might not always have a greater competitive advantage over their non-invasive native counterparts [29]–[32]. Abrahamson and Gadgil (1973) demonstrated that late successional species did not have higher reproductive effort compared to early successional species. In addition, Sans et al. (2004) compared three congeners including two exotic invasive and one native species and found that the reproductive effort of the native species was the highest in all treatments. High reproductive effort is usually associated with stressed environments [33]–[35]. Thus, plants in stressed environments should invest more resources into reproductive and not vegetative structures. However, studies have provided evidence that reproductive effort did not hold constant with changes in the different environments [36]–[39]. Hence, there are still inconsistencies in studies of reproductive effort.

In a previous study we compared one early and one late successional species by ANCOVA to distinguish allometric exponents in response to environmental treatments that showed higher plasticity of the late successional species [40]. However, the results are limited in extrapolation to broader patterns of succession. The present study includes two early and two late successional species, because the trends of all four species provide more information on phenotypic plastic responses of early and late successional species than previous study. We used SMATR (Standardised Major Axis Tests & Routines, [41] to evaluate allometric relationships between biomass and other plant measurements for increased statistical inference that our first study could not provide [40].

By comparing morphological traits (in relation to resource partitioning) and reproductive effort, we conducted a controlled (including nutrients, water, and density) greenhouse experiment comparing early successional species (*Agriophyllum squarrosum* and *Corispermum macrocarpum*) that colonize a resource-poor environment, with later successional species (*Chenopodium acuminatum* and *Salsola collina*) that colonize a higher- resource environment and replace the early successional communities, in Horqin sandy land. We tested three hypotheses: (1) species that exhibit different invasion scenarios are better adapted to different successional stages; (2) late successional species have higher reproductive effort; and, (3) environmental stresses do not uniformly increase reproductive effort.

## Materials and Methods

### Study area

This study was conducted in mobile and stabilized sand dunes of south-western Horqin Sandy Land, Inner Mongolia, China (42°55′N, 120°42′E; elevation approx. 345 m). The climate of this region is semi-arid. The average annual temperature is 6.8°C with monthly averages ranging from a minimum of −13.1°C in January to a maximum of 23.7°C in July. The average annual precipitation is 360 mm with 75% of this in the growing season of June–September [42]–[44].

### Study species

Four annual forbs were selected from the family Chenopodiaceae: two early successional status species (*Agriophyllum squarrosum* and *Corispermum macrocarpum*) of mobile sand dunes, and two later successional species (*Chenopodium acuminatum* and *Salsola collina*) of stabilized dunes and cropland, which are commonly found in abandoned habitats [45], often in inter-dune lowland and cropland [46], which play an important role in restoration succession in degraded land in Horqin Sandy Land. *A. squarrosum* and *C. macrocarpum* are adapted to extremely degraded sandy soil, and are also the pioneer species in this habitat. Later successional dunes become stabilized by shrubs such as *Artemisia halodendrom* Turcz. ex Bess and *Salix gordejevii* Chang et Skv., and *C. acuminatum* and *S. collina* colonize with the gradual disappearance of *A. squarrosum* and *C. macrocarpum* [42], [46], [47].

### Experimental design

All species' seeds, which were separately collected from a population for each species within the early and later successional community, were sown on May 9^th^ 2007 in plastic plates with sandy soil obtained from the severely degraded sand dune. The seedlings were transferred into 13.8-cm-radius×26.5-cm-deep plastic pots with the same sandy soil after 4 weeks of sowing (i.e. two-leaf stage). Treatments were imposed 2 weeks after planting. Because of the increasing soil nutrient, soil water and population density during this ecological succession, each of four species was treated with different levels of soil nutrients, soil water and population density. Each factor had two levels: N+: High nutrient level, 20 g of slow-release fertilizer (Osmocote, containing N 14%,P 14% and K 14% and microelements, The Scotts Company), mixed with sandy soil at the beginning of the experiment (total nitrogen concentration was nearly 200 mg/kg soil, similar to stabilized dunes, which ranged from 200 to 300 mg/kg soil) [48]; N–: Low nutrient level, no nutrients added (the total nitrogen concentration of sandy soil ranged from 49 to 53 mg/kg soil, measured using the Kjeldahl method) [49]–[51]; W+: High water level, equivalent to 400 mm of rainfall during the growing season applied as 530 mL of water every 3 days; W–: Low water level, equivalent to 200 mm of rainfall during the growing season applied as 265 mL of water every 3 days (which is approximately equal to the rainfall during growth season in Horqin Sandy Land); D+: High density level, six plants in each pot arranged is an equilateral hexagonal fashion (which was equivalent to 100 plants/m^2^, similar to the density in a crowded environment); D–: Low density level, three plants in each pot arranged by equilateral triangular fashion (which was equal to 50 plants/m^2^, similar to the density in normal environment). A full-factorial design of the 32 possible combinations of four species, two levels of soil nutrients, water, and population density was constructed. Each treatment combination had 102 plants (34 pots in each low population density treatment and 17 pots in each high population density). All pots were placed in a naturally ventilated greenhouse in order to reduce differences between the inside and outside of the greenhouse. In order to eliminate positional effects, the pots in each combination were regarded as a group and placed together, group positions were rotated every two weeks.

### Measurements

The plants were harvested from 13^th^ to 19^th^ September, when the reproduction of plants was completed [52], and 12 to 20 intact plants from each treatment were randomly selected for measurements. The roots were washed gently until no soil was visible. Each plant was separated into vegetative organs (including roots, stems, and leaves) and reproductive organs. These organs were oven dried at 80°C to a constant mass to determine the respective dry mass. The shattered seeds on the ground were not collected at the final harvest (seed losses were not more than 0.1% of reproductive biomass). The following traits were recorded for individual plant: absolute height, number of primary branches, number of secondary branches, vegetative biomass (including roots, stems, and leaves), reproductive biomass, total biomass, reproductive effort (reproductive biomass/total biomass). The phenotypic plasticity index (PPI, (Maximum mean-minimum mean)/maximum mean) was calculated for each trait.

### Statistical analysis

A four-way factorial analysis of variance (ANOVA) was performed to test the effects of species (S), nutrients (N), water (W) and density (D) on plant traits with SPSS statistical software (version 15.0) (SPSS Inc., Chicago, Illinois). The phenotypic plasticity was quantitatively estimated by the slope of norm of reaction. Interaction between species and environments showed the difference of phenotypic plasticity between different species for each trait.

Regressions of reproductive biomass (*Y*) on vegetative biomass (*X*) were conducted to characterize the allometry of reproduction in different environmental conditions. The allometric relationship can be described by log *y* = log *b*+a * log *x*, where *a* is the scaling exponent (slope) and *b* is the allometric coefficient or “scaling factor” (*y* intercept). Differences in shifts of the slope and in elevation of slopes (*y*-intercept) were assessed using standardized major axis regression (SMA, also known as reduced major axis, RMA) using the SMATR package in R software [41], [53]. Standardized major axis regression (SMA) is commonly required for allometric studies [54], [55]. SMA analyses are appropriate for summarizing the relationship between two variables in terms of a single slope [56]. In SMATR heterogeneity between SMA slopes is tested via a permutation test. Differences in SMA slope, elevation (intercept) and plant size are estimated [55].

## Results

### Phenotypic plasticity of plant morphological traits

**Figure 1:**
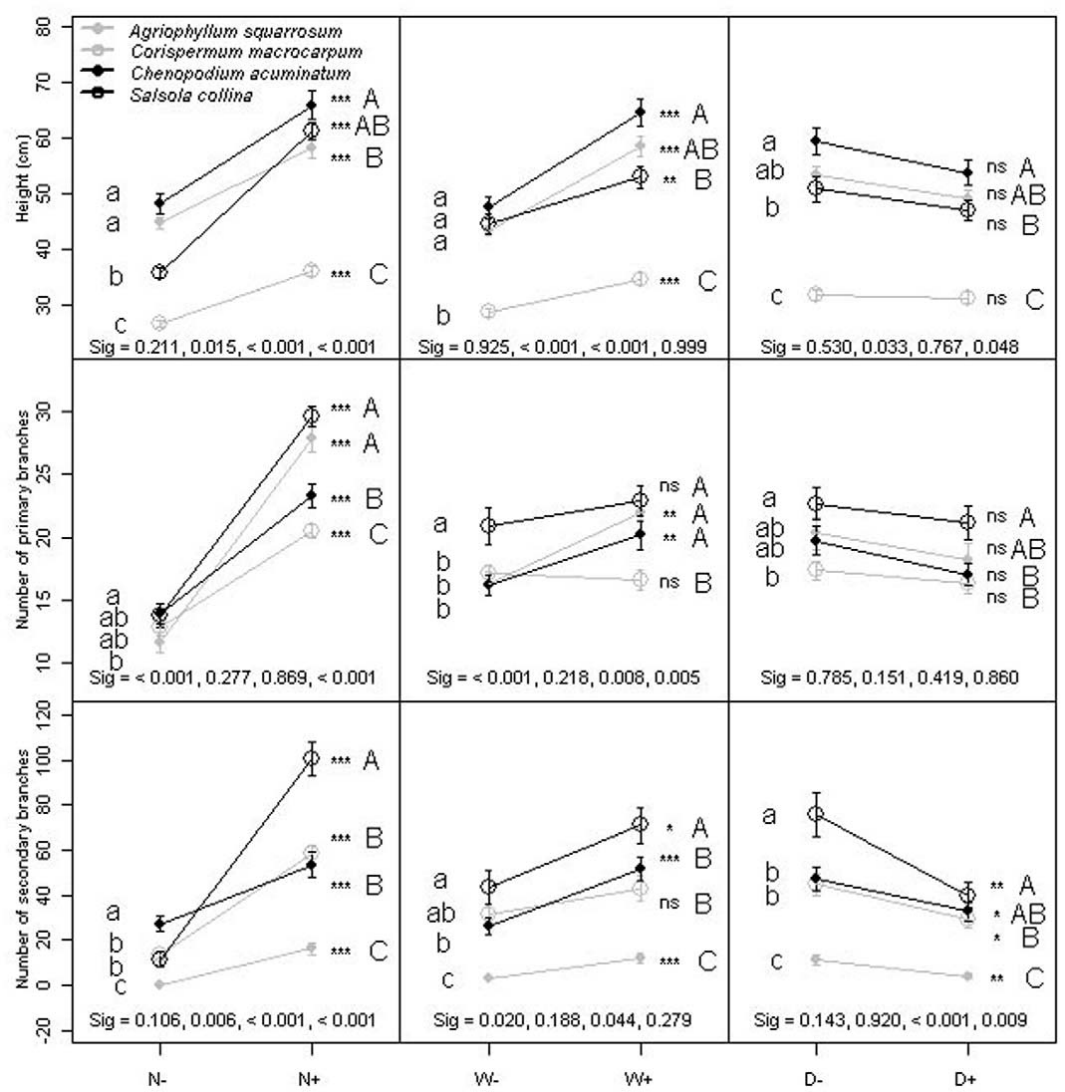
Norm of reaction for four species at two soil nutrients (N), water (W) and population density (D) levels on morphological traits; + and − indicate two levels of factor; The data represent means ± SD of average across treatments; ns denote not significance (*P*>0.05), asterisks denote significance at *P*<0.05 (*), *P*<0.01 (**) and *P*<0.001 (***) for each species in different level environmental treatments; the same lower case letters within columns are not significantly different at *P*<0.05 in low level environmental treatment for four species; the same capital letters within columns are not significantly different at *P*<0.05 in high level environmental treatments for four species. Sig, the *P* value of interactions between traits and environmental factors when compared between *C. acuminatum* and early succession species (*A. squarrosum* and *C. macrocarpum*) and between *S. collina* and early succession species (*A. squarrosum* and *C. macrocarpum*).

Four way factorial ANOVA showed that morphological traits varied significantly between the four species and among treatments (all *P*<0.001, Table 1). All morphological traits increased with high soil nutrient and water availability for the four species (all *P*<0.001, Table 1). Only the number of secondary branches for individual species was significantly affected by population density, and decreased with increasing population density (Table 1; Fig. 1). Interactions between species and environmental factors (S×N, S×W and S×D) varied across morphological traits, which imply shifts in plasticity.

**Table 1:** Analysis of variance for plant traits of four species under soil nutrients, water, and population densities.

	d.f.	Height	Number of primary branches	Number of secondary branches	Total biomass	Reproductive effort
Species (S)	3	160.51***	14.53***	73.40***	20.86***	470.42***
Nutrients (N)	1	366.64***	498.54***	342.69***	236.08***	350.69***
Water (W)	1	168.53***	20.52***	48.37***	124.90***	64.25***
Density (D)	1	23.82***	15.36***	66.54***	79.48***	0.01
S * N	3	14.03***	16.10***	43.50***	14.62***	3.62*
S * W	3	10.4***	5.86***	2.02	3.18*	1.27
S * D	3	2.10	0.83	6.14***	2.51	1.13
N * W	1	42.23***	8.72**	11.52***	72.69***	11.31***
N * D	1	0.26	0.52	45.71***	29.06***	0.12
W * D	1	0.80	0.25	0.34	1.85	0.96
S * N * W	3	6.47***	5.90***	3.83**	2.59	6.55***
S * N * D	3	6.75***	1.11	14.13***	8.39***	0.79
S * W * D	3	1.34	2.86*	2.76*	0.67	1.61
N * W * D	1	2.41	0.27	1.79	7.43**	0.11
S * N * W * D	3	10.30***	3.50*	3.53*	4.32**	2.88*

*F*-values are presented.

* refers to significance at *P*<.05;

** to significance at *P*<.01;

*** to significance at *P*<.001.

The *C. acuminatum* height was 65.8 cm, which was highest among the four species in the high soil nutrient treatment (*P* = 0.05 level). *S. collina* height significantly increased 0.71 times (the phenotypic plasticity index was 0.42) in response to soil nutrients (*P*<0.001). The significant interactions between species and soil nutrients showed that the plasticity of *S. collina* was greater with higher nutrients than *A. squarrosum* and *C. macrocarpum* (both *P*<0001, Fig. 1). In response to soil water, *C. acuminatum* (PPI = 0.26) had the same plasticity as *A. squarrosum* (PPI = 0.26) (*P* = 0.925), but had higher plasticity than *C. macrocarpum* (PPI = 0.17) (*P*<0001). In the high soil water treatment, there was no significant difference in height performance between *S. collina* and *A. squarrosum*, but the value of *S. collina* was significantly higher than *C. macrocarpum*. Although the interaction between species and population density was not significant, *C. acuminatum* (PPI = 0.09) and *S. collina* (PPI = 0.08) both had higher plasticity in height than *C. macrocarpum* (PPI = 0.02) by comparing between two species (*P* = 0.033 and 0.048, respectively). The heights of *C. acuminatum* and *S. collina* were higher than *C. macrocarpum*, but not higher than *A. squarrosum* in the high population density (Fig. 1).

The numbers of primary branches in high nutrient soil for *A. squarrosum* (PPI = 0.58) and *S. collina* (PPI = 0.53) were higher than *C. macrocarpum* (PPI = 0.37) and *C. acuminatum* (PPI = 0.40), as was the plasticity in response to soil nutrients (all *P*<0.001). The increase in numbers of primary branches for *A. squarrosum* (PPI = 0.25) and *C. acuminatum* (PPI = 0.20) was significant in response to soil water (*P* = 0.002 and 0.006, respectively), while the numbers of primary branches of *C. acuminatum* and *S. collina* were not higher than *A. squarrosum*, but higher than *C. macrocarpum*. However, there was no difference in plasticity of plant height among four forbs in response to population density (*P* = 0.480).

*S. collina* had a higher number of secondary branches and higher plasticity than the other three species, (*P* = 0.006, <0.001 and <0.001, respectively for plasticity). The number of secondary branches of *C. acuminatum* in high soil nutrients was not higher than *C. macrocarpum*, but higher than *A. squarrosum*. The plasticity of number of secondary branches for the two late successional species increased (by 27.5 and 25.6 branches respectively) in response to soil water compared to *A. squarrosum* (*P* = 0.020 and 0.044, respectively). The decreases in number of secondary branches of all four species were significant in response to density (*P* = 0.004, 0.011, 0.031 and 0.002, respectively), but the value of *S. collina* was higher than both late successional species, and value of *C. acuminatum* was higher than *A. squarrosum* (Fig. 1).

### Plasticity of biomass traits and reproductive effort

**Figure 2:**
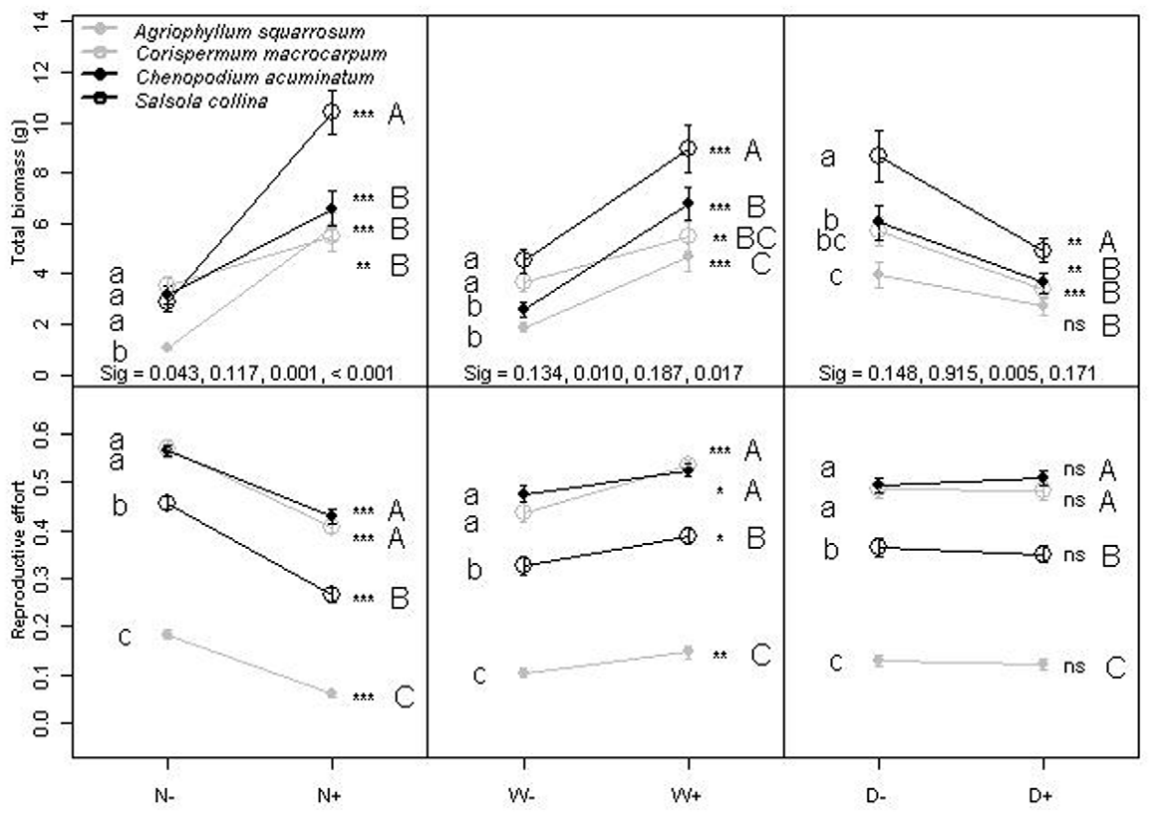
Norm of reaction for four species at two soil nutrients (N), water (W) and population density (D) levels on total biomass and reproductive effort of four species; + and − indicate two levels of factor. The data represent means ± SD of average across treatments; Asterisks denote significance at *P*>0.05 (ns), *P*<0.05 (*), *P*<0.01 (**) and *P*<0.001 (***) for each species in different level environmental treatment; the same lower case letters within columns are not significantly different at *P*<0.05 in low level environmental treatments for four species; Same capital letters within columns are not significantly different at *P*<0.05 in high level environmental treatment for four species. Sig, the *P* value of interactions of between total biomass and environmental factor when compared between *C. acuminatum* and early succession species (*A. squarrosum* and *C. macrocarpum*) and between *S. collina* and early succession species (*A. squarrosum* and *C. macrocarpum*).

The total biomass was significantly affected by all three environmental factors as well as their interactions except for W×D. Total biomass was significantly affected by S×N and S×W interactions (Table 1). The biomass of *S. collina* was 8.96 g in high soil nutrients and increased 2.63 times compared to low nutrients (Fig. 2). In response to soil nutrients, the phenotypic plasticity indices of *A. squarrosum*, *C. macrocarpum*, *C. acuminatum* and *S. collina* were 0.82, 0.35, 0.52 and 0.72, respectively. Except for similar plasticity between *C. acuminatum* and *C. macrocarpum* (*P* = 0.177), the late successional species were more plastic in response to soil nutrients than both early successional species (Fig. 2). Biomass values in high soil water and plasticity of late successional species (the phenotypic plasticity indices of *A. squarrosum*, *C. macrocarpum*, *C. acuminatum* and *S. collina* were 0.60, 0.34, 0.62 and 0.50, respectively.) were higher than early successional species (Fig. 2). The plasticity of total biomass was not significantly different among four forbs in response to population density except between *S. collina* and *A. squarrosum* (*P* = 0.005). In high population density, the value of *S. collina* was higher than the other three species, and the value of *C. acuminatum* was not significantly higher than early successional species (*P*>0.05 level) (Fig. 2).

**Figure 3:**
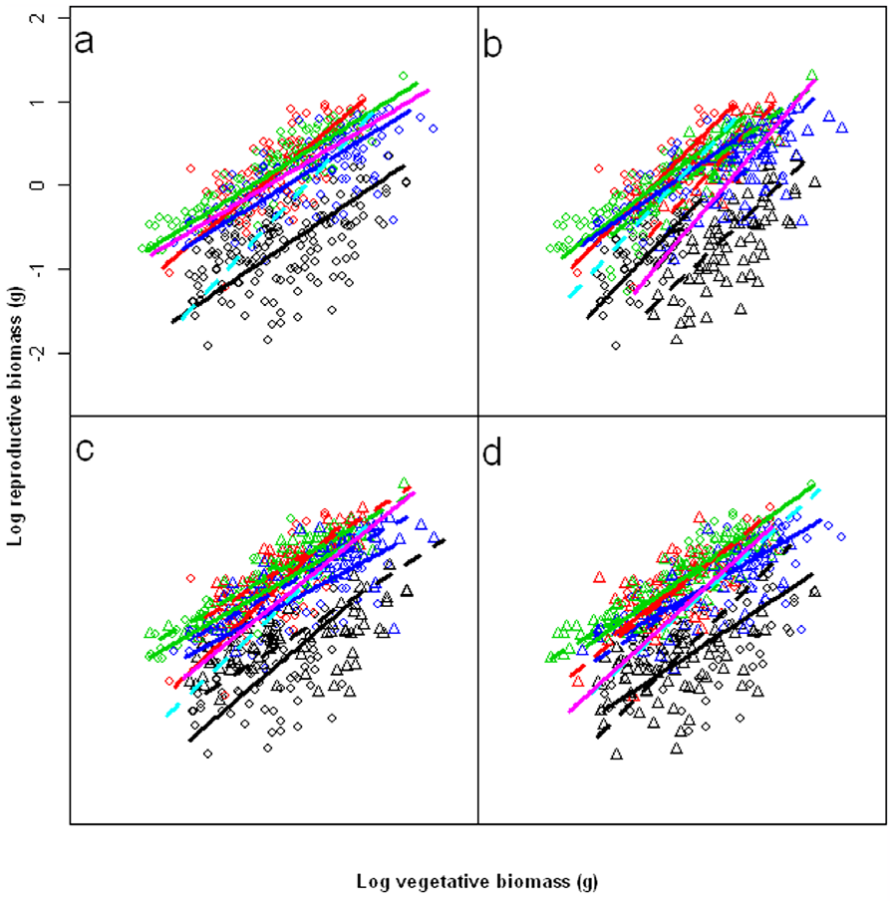
Log10-log10 plots showing the relationship between reproductive biomass and vegetative biomass of (a) *A. squarrosum* (black), *C. macrocarpum* (red), *C. acuminatum* (green) and *S. collina* (blue); and SMA fit line for combining all early successional species (cyan dashed line) and combining all late successional species (magenta solid line). For b (soil nutrient treatments), c (soil water treatments), and d (population density treatments), low environmental level (circle points and solid line) and high soil nutrient treatment level (triangle points and dashed line), black, red, green and blue, respectively for *A. squarrosum*, *C. macrocarpum*, *C. acuminatum* and *S. collina*, and SMA fit line for combining all low environmental levels (cyan dashed line) and combining all high environmental levels (magenta solid line).

Reproductive effort of late successional species was not always higher than early successional species (Fig. 2). Only the interaction between species and soil nutrients on reproductive effort was significant (Table 1). The values of *C. acuminatum* and *C. macrocarpum* were highest, the value of *S. collina* was intermediate, and the value of *A. squarrosum* was lowest across all high level treatments (*P* = 0.05). Reproductive biomass was positively correlated with vegetative biomass in all groups (Table 2, Fig. 3). Individual SMA (standardized major axis) slopes varied significantly among four species. When combining the same successional status species, individual SMA slopes varied significantly between the two successional status groups (*P*<0.001) (Table 2, Fig. 3a). When comparing individual species, soil nutrient and soil water treatments had a significant affect on the individual SMA slopes or *y*-intercepts. However the population density treatment only affected the plant size (shift along on the common slope), and did not have an effect on the slopes or *y*-intercepts (Table 3). Fig. 3(b, c, d) showed the result of combining all species in a common treatment. Individual SMA slopes varied significantly between the two soil water treatments (*P* = 0.030) (Table 2, Fig. 3c). Individual SMA slopes did not vary significantly between the two soil nutrient groups (*P* = 0.195) (Table 2, Fig. 3b). There was a significant difference in *y*-intercepts among groups (*P*<0.001), as well as significant differences in group shifts along a common SMA (*P*<0.001), indicating that shifts in vegetative biomass resulted in associated shifts in reproductive biomass. High nutrient plants had higher reproductive biomass and vegetative biomass than low nutrient plants (Fig. 3b). Individual SMA slopes did not vary significantly between two population densities (*P* = 0.715) (Table 2). There was no significant difference in *y*-intercepts among the SMA slopes (*P* = 0.390), allowing a common SMA to be fitted to examine group shifts. Significant differences in population density shifts were evident along the common SMA (*P*<0.001), with low population density plants having higher reproductive biomass and vegetative biomass than high population density (Table 2, Fig. 3d).

**Table 2:** Results of standardized major axis regression (SMA) analysis of pairwise combinations of reproductive biomass and vegetative biomass for combining all species in different treatments.

Group	Level	*n*	*r^2^*	*p*	Slope	Intercept	Slope homogeneity (*P*)	Shift in elevation (*P*)	Shift along slope (*P*)
Succession	Early	280	0.161	**<0.001**	1.490	−0.651	**<0.001**	**<0.001**	**<0.001**
	Late	268	0.627	**<0.001**	0.819	−0.092			
Nutrient	Low	278	0.434	**<0.001**	1.480	−0.138	0.195	**<0.001**	**<0.001**
	High	270	0.182	**<0.001**	1.623	−0.884			
Water	Low	271	0.205	**<0.001**	1.308	−0.455	**0.030**	0.072	**<0.001**
	High	277	0.276	**<0.001**	1.112	−0.320			
Density	Low	266	0.297	**<0.001**	1.222	−0.423	0.715	0.390	**<0.001**
	High	282	0.236	**<0.001**	1.256	−0.388			

Significant results (P<0.05) are shown in bold.

**Table 3:** Results of standardized major axis regression (SMA) analysis of pairwise combinations of reproductive biomass and vegetative biomass for each species in different treatments.

Species	Nutrient			Water			Density		
	*P*1	*P*2	*P*3	*P*1	*P*2	*P*3	*P*1	*P*2	*P*3
*A. squarrosum*	0.369	**<0.001**	**<0.001**	**<0.001**	**0.002**	**<0.001**	0.744	0.552	**0.016**
*C. macrocarpum*	0.223	**<0.001**	**<0.001**	**<0.001**	**<0.001**	0.004	0.133	0.589	**<0.001**
*C. acuminatum*	0.097	**<0.001**	**<0.001**	0.225	**<0.001**	**<0.001**	0.223	0.763	**0.002**
*S. collina*	**<0.001**	**<0.001**	**<0.001**	0.739	**<0.001**	**<0.001**	0.232	0.430	**0.015**

*P*1 is the test of slope homogeneity, *P*2 is the test of shift in elevation, *P*3 is the test of Shift along slope. Significant results (P<0.05) are shown in bold.

## Discussion

As with previous studies [57]–[60], the response of morphological traits to environmental variation was investigated here. Our experiment clearly demonstrated that both early and late successional species exhibited significantly taller plants, more branches and more biomass under increased nutrients and water and decreased density [52], [61], [62].

Late successional sand dunes have higher soil water holding capacity and higher soil moisture, and they also have higher nutrients [51], [63]. The late successional species had greater growth responses to higher soil nutrients and water. The height of the two late successional species in the high resource treatments was not significantly higher than *A. squarrosum*. It may be related to the habitat of *A. squarrosum*, which is a pioneer species on the mobile dune, where the competition of intra- and interspecific competition is weak [61], [64], [65]. *C. macrocarpum* had lower values for plasticity than late successional species, except for secondary branch production. On the whole, late successional species had higher values, which indicates they exhibit the traits of a Jack-of-all-trades, showed better performance in favorable environments. They also fit another idealized scenario Master-of-some, as they are better able to increase fitness in favorable environments, than early successional species in response to environmental factors [66].

Thus, the average performance of both late successional species was always higher than both early successional species, and the effect of interactions between succession and environmental factors also were significant. Based on our study, during the succession of degraded sandy sites, late successional species were better able to increase fitness for some traits or maintain fitness for other traits. This may be related to the higher resource levels of the stabilized dunes. The results of this study are in agreement with the first hypothesis; patterns of invasive species' plasticity can be applied to succession.

An earlier study [30] indicated that the reproductive effort of exotic species was lower than that of the native plants in all treatments, which conflicts with other reports [27], [67] that invasive species exhibit higher reproductive effort. The reason for this inconsistency could be because species [67] or development time [68]. Stastny et al (2006) were compared between populations of the same species, and the same patterns may not hold true between different species, since each species will have its own intrinsic regulation of reproductive effort [30]. Because under high resource levels plants may postpone reproduction, increased resource levels can give the impression of reduced reproductive effort if plants do not have time to complete their life cycles [68]. More investigations on different species of plants, with completed reproduction, are therefore necessary in order to fully understand the mechanism of reproductive effort. However, in a successional study, the late successional species often had high reproductive effort [69]. The mid-late successional species allocated most resources to reproductive effort in order to continue the population [70]. In this study, late successional species have higher reproductive effort than *A. squarrosum*. However the ecological amplitude of *C. macrocarpum* is broad, it produces a lot of seeds to colonize in mobile, semi- mobile and semi-fixed dunes. The average performance showed that late successional species had higher reproductive effort, but at the species level, the second hypothesis was not true, late successional species did not have higher reproductive effort than early successional species. This may be related to the fact that *C. macrocarpum* can also be an early successional species.

Reproductive effort can vary with different environmental factors [71], and either increase [33] or decrease [72] in response to an increasing plant density. In the present study, density had no effect on biomass allocation and allometry of reproductive biomass. The plants exhibited similar biomass allocation ratios across all densities. Reproductive effort greatly depends on nutrients and water. The scaling exponent (slope) between vegetative and reproductive biomass was consistent, but the allometric coefficient and plant size varied with different soil nutrients. So under high soil nutrient, plants allocated less biomass to reproductive organs than to other organs. Besides plant size, soil water had significant effects on the scaling exponent, which was the allometric coefficient between vegetative and reproductive biomass in this study, so with high soil water availability, plants allocated less biomass to other organs than to reproductive organs at the same plant size. Previous reports showed that plants increase reproduction in response to environmental stress [33]–[35], or that plants have similar levels of reproductive output in resource-poor environments. This means that the ability of plants to maintain fecundity at low resource levels or to “make the best of a bad job” [73]. In contrast, our study supports the hypothesis that plants do not always increase reproductive effort for any environmental stressors. Subtraction of nutrients can increase reproductive effort, while addition of water can increase reproductive effort, and altering density may not affect reproductive effort or the allometry of reproductive biomass.

In conclusion, the plasticity patterns were related to successional status. For late successional species, it was a complex of Master-of-some and Jack-of-all-trades. Late successional species had higher performance in high soil nutrients and water or higher plasticity in response to soil nutrients and water than early successional species. Our results show that reproductive effort for late successional species was not always higher than for each early successional species. In contrast to the effect of water, the addition of nutrients lead to a decline in reproductive effort, while density did not have any effect on reproductive effort. Early successional species were in general less plastic in their response, perhaps a reflection of their adaptation to resource-poor early successional soil.
